# 2016 ISCB Overton Prize awarded to Debora Marks

**DOI:** 10.12688/f1000research.9158.1

**Published:** 2016-07-05

**Authors:** Christiana N. Fogg, Diane E. Kovats

**Affiliations:** 1 Freelance Science Writer, Kensington, MD, USA; 2 International Society for Computational Biology, Bethesda, MD, USA

**Keywords:** ISCB, Overton Prize, Debora Marks, Dr. G. Christian Overton, systems biology

## Abstract

The International Society for Computational Biology (ISCB) recognizes the achievements of an early- to mid-career scientist with the Overton Prize each year. The Overton Prize was established to honor the untimely loss of Dr. G. Christian Overton, a respected computational biologist and founding ISCB Board member. Winners of the Overton Prize are independent investigators in the early to middle phases of their careers who are selected because of their significant contributions to computational biology through research, teaching, and service. 2016 will mark the fifteenth bestowment of the ISCB Overton Prize.  ISCB is pleased to confer this award the to Debora Marks, Assistant Professor of Systems Biology and director of the Raymond and Beverly Sackler Laboratory for Computational Biology at Harvard Medical School.

ISCB is pleased to recognize Debora Marks, Assistant Professor of Systems Biology and director of the Raymond and Beverly Sackler Laboratory for Computational Biology at Harvard Medical School. She will accept this honor and present a keynote talk at ISMB 2016 in Orlando, Florida, on Sunday, July 10th.

As a child and young adult, Marks never considered becoming any sort of scientist. She was fairly confident that she was either going to travel in time around the universe in the tardis with Dr. Who, or be a professional political protester and save the world. However, math was a constant that captured Marks’s attention since she was a little girl and she recalls spending far too much time with math puzzle books, “math was one of the only activities that forced me to focus and calmed my brain.”

After school, Marks went to study medicine at the University of Bristol (in England) but left now with some regret after her 2nd MBChB degree in 1980 as she was “more interested in theatre and politics than Latin names for bones”. Many years later, after babies and a variety of interesting jobs, Marks felt the pull back to academia and her first love, mathematics, and went on to complete her BSc Hons degree in mathematics in 1993 at Manchester University. She recalled the focused scope of her mathematics studies during a time when students were not allowed to attend other courses, and interdisciplinary studies were not yet *en vogue*. “In England, when you did a math degree, it was a math degree and you were not supposed to dilute it with ‘lower value’ subjects like biology, computer science or even physics. I did however manage to attend an odd course on chaos and fractals that sparked my interest in the intersection of math and biology that continues to drive me today.”

Marks considers her introduction to computational biology somewhat unorthodox and recalled, “I came to computational biology by jumping in the deep end. After my math degree I won an award from the Wellcome Trust to research drug design for *Leishmania* and trypanosomiasis. I was given a Silicon Graphics machine and told to get to work. I hadn’t got a clue. I’d never used a computer. Because I had a math degree, they thought I was a computer scientist of sorts.”

In the wake of the Human Genome project, Marks went on to get a bioinformatics position at Harvard at a time when interest in the potential for computation in biological research intensified. MicroRNAs first captured her attention in mid-2000, and her work on these was eventually submitted as a PhD thesis in 2010 at the Humboldt University in Berlin under the guidance of Reinhard Heinrich and, after Heinrich’s untimely passing, completed with Hanspeter Herzl as thesis mentor.

She recalled, “I accidently read an article in a biology journal lying around about what was then a semi-obscure discovery about small RNAs regulating development in worms. I couldn’t stop thinking about it. Do these little RNAs stick to more than one gene? Maybe humans have them?” MicroRNAs were obscure at the time, only two were known, not the category. The floodgates only opened after the discovery of tens (at the time) of the now named “microRNAs,” nearly identical in sequence across worms, flies and humans, published in three back-to-back papers in October 2001 and suggesting strong selection across many species. “So, what are they doing in all these organisms?” As others identified more and more of these microRNAs, it became obvious to Marks that a way was needed to find out what processes they regulated. Unlike the much more complicated task of identifying targets of proteins, the chemistry of base-pairing suggested an obvious way to explore what microRNAs might stick to.

Marks was the first, concurrently with the Cohen and Bartel labs, to publish genome-wide targeting by microRNAs, first in fly, then in human, having developed the miRanda algorithm that is still used today for target prediction. “Although many groups have now published papers on how to discover microRNA targets,” Marks said “At best, the science of microRNA target prediction is still imprecise, presenting an unmet challenge for the computational community.” These early papers highlighted the potential genomic scope of microRNA targeting across large pools of mRNAs and their many-to-many, cooperative and combinatorial regulation of protein expression, something we now take for granted. Struck by these indications of potential system-wide effects, Marks undertook work to investigate the function of small RNAs in the context of the cellular environment by using mathematical modeling, re-analysis of previously published experiments, and additional *in vitro* experiments.

She made several key findings that included demonstrating that mRNA half-life influences the effects of microRNA and siRNA targeting for thousands of gene targets and that mRNA and microRNA abundance impacts microRNA targeting (now thought of as the mRNA sponge effect, and, controversially, “ceRNAs”). She also showed that introducing siRNAs or microRNAs into cells results in attenuation of endogenously regulated genes. Marks explained, “This is a really important consideration for the interpretation of gene knock-down experiments and for therapeutic uses of small RNAs.” Her more recent work showed that for a given level of protein, adding microRNA regulation can reduce protein noise or fluctuations, especially for transcripts with low expression.

Quite by chance, Marks’s postdoctoral work shifted sharply away from microRNAs to the field of *ab initio* 3D structure prediction of proteins. Together with Chris Sander, they revisited an older idea that had been advanced independently by the groups of Sander, Neher and Taylor in the mid-1990s of using covariation of residues in proteins across evolution to identify residues that might be in contact in 3D. They reasoned that if these inferred contacts were accurate enough, one should be able to fold a protein sequence using simple methods such as distance geometry and restrained molecular dynamics. The key advances were to use a statistically global model of covariation across the sequences that removes transitive correlations in the data, by using a probability model for entire proteins in the sequence family, not unlike a suggestion made by Gary Stormo and Alan Lapedes in 1999. She said, “We stumbled across statistical physics models that are used to determine inhomogeneous interactions in Ising models from observed data that contain transitive correlations. Listening to the team of Riccardo Zecchina, including Andrea Pagnani and Martin Weigt, in Torino, Italy made us think that their approaches to the analysis of correlated mutations could be important for the 3D structure-from-sequence problem. Working with their team in collaboration, I set off in the spring of 2010 to see if the maximum entropy method could work to find truly interacting co-evolved residues. If the computation was correct then co-evolved residue pairs should match contacts in known 3D structures, and they did nearly to the ceiling. I then cajoled a friend, Lucy Colwell who, like me, was also a recent graduate, to join the project. What fun we had! Well, that is until it was time to try and publish it. Reviewers found it difficult to believe, but eventually we published the results at the end of 2011, and the EVfold community has grown ever since.” Marks explained, “The method is very democratic. It is fast and can be run on a laptop, even the folding, and relies only on gene sequences. Immediate applications were to proteins that are challenging experimentally such as large membrane proteins and to protein-protein complexes. More than five of the transmembrane proteins have since been crystallized and agree well with the predicted structures, such as the adiponectin receptor.” “A very effective mini-CASP,” she added with a smile.

After the protein folding breakthrough in the fall of 2010, implementation of similar methodology led her to the solution, published in 2016, of another hard and unsolved problem in computational biology, that of computing RNA 3D structures and of RNA-protein complexes just from sequence information.

Currently, Marks has a newly formed lab at Harvard Medical School and is building the Raymond and Beverly Sackler Laboratory for Computational Biology. She is continuing the “3D from sequence” work, including new types of biomolecular interactions and their conformational flexibility and dynamics. The Marks lab is also going back to math and developing the core algorithms for sequences, and model inference for multidimensional biological data. At the same time, Marks is branching out with new applications that include the challenge of predicting the effects of genetic variation on disease risk and drug response, especially combinations of events, and particularly in antibiotic resistance “It may seem we are promiscuous in our choice of biological questions, but the underlying thread is one of solving problems that are hard to solve experimentally. One far-reaching question that I am increasingly less embarrassed to admit being interested in is: What makes us all different? With genomes in hand, surely we can now find out how much is nature, how much is nurture and how much is stochastic?”

Marks is grateful to her mentors, her “wonderful” Systems Biology department and the Raymond and Beverley Sackler Foundation who have supported her throughout her unusual career path and feels greatly honored to be recognized with the Overton Prize. She is especially thankful to her new group members and her long-time scientific collaborator Chris Sander. She said, “I want to share the prize in spirit with all those who have tolerated and encouraged me despite the odds.”

Marks’s final message to young scientists (young in spirit, that is) is to “go big, go risky, and learn statistics (!)”

